# Tricyclo­hex­yl(3,5-dibromo-2-hy­droxy­benzoato-κ*O*)tin(IV)

**DOI:** 10.1107/S1600536811020496

**Published:** 2011-06-11

**Authors:** Xi-Cheng Liu, Wen-Tao Jiang, Peng-Cheng Shan, Wen-Chao Ding

**Affiliations:** a Department of Chemistry, Qufu Normal University, Qufu 273165, People’s Republic of China

## Abstract

In the title compound, [Sn(C_6_H_11_)_3_(C_7_H_3_Br_2_O_3_)], the Sn atom is four-coordinate and possesses a distorted Sn(C_3_O) tetra­hedral geometry, with Sn—C bond lengths in the range 2.132 (6)–2.144 (6) Å and with Sn—O = 2.086 (4) Å. The uncoordinated carboxyl­ate O atom forms a weak contact with the Sn atom, with an Sn⋯O separation of 2.962 (2) Å.

## Related literature

For background information on organotin carboxyl­ate compounds, see: Davies *et al.* (2008 ▶); Tian *et al.* (2005 ▶). For related structures, see: Baul *et al.* (2001 ▶); Rauf *et al.* (2008 ▶); Smith *et al.* (1986 ▶); Song *et al.* (2002 ▶); Wang *et al.* (2007 ▶); Willem *et al.* (1998 ▶).
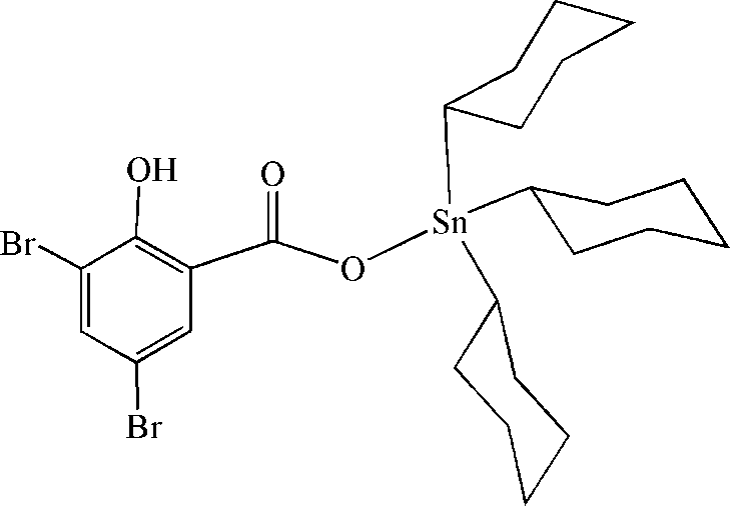


## Experimental

### 

#### Crystal data

[Sn(C_6_H_11_)_3_(C_7_H_3_Br_2_O_3_)]
*M* *_r_* = 663.05
Monoclinic, 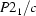

*a* = 9.4912 (13) Å
*b* = 17.640 (2) Å
*c* = 18.0655 (18) Å
β = 117.200 (5)°
*V* = 2690.1 (5) Å^3^
*Z* = 4
Mo *K*α radiation
μ = 3.94 mm^−1^
*T* = 295 K
0.20 × 0.20 × 0.10 mm

#### Data collection

Bruker SMART APEX area-detector diffractometer
Absorption correction: multi-scan (*SADABS*; Bruker, 2002 ▶) *T*_min_ = 0.506, *T*_max_ = 0.694
19711 measured reflections
5000 independent reflections
3416 reflections with *I* > 2σ(*I*)
*R*_int_ = 0.050

#### Refinement

*R*[*F* ^2^ > 2σ(*F* ^2^)] = 0.048
*wR*(*F* ^2^) = 0.136
*S* = 1.03
5000 reflections
280 parameters
20 restraints
H-atom parameters constrained
Δρ_max_ = 1.18 e Å^−3^
Δρ_min_ = −0.93 e Å^−3^

### 

Data collection: *SMART* (Bruker, 2002 ▶); cell refinement: *SAINT* (Bruker, 2002 ▶); data reduction: *SAINT*; program(s) used to solve structure: *SHELXS97* (Sheldrick, 2008 ▶); program(s) used to refine structure: *SHELXL97* (Sheldrick, 2008 ▶); molecular graphics: *XP* (Bruker, 2002 ▶); software used to prepare material for publication: *SHELXL97*.

## Supplementary Material

Crystal structure: contains datablock(s) global, I. DOI: 10.1107/S1600536811020496/bh2352sup1.cif

Structure factors: contains datablock(s) I. DOI: 10.1107/S1600536811020496/bh2352Isup2.hkl

Additional supplementary materials:  crystallographic information; 3D view; checkCIF report

## Figures and Tables

**Fig. 1.**
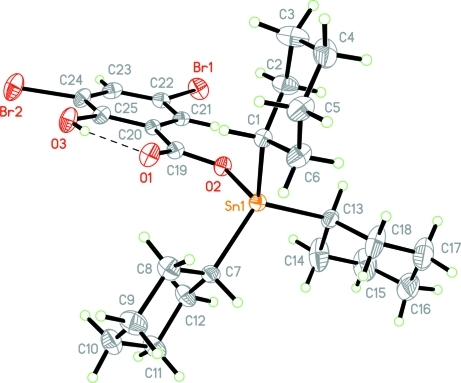
The structure of the title compound, with displacement ellipsoids drawn at the 30% probability level.

## supplementary crystallographic information

### Comment

Organotin carboxylates form an important class of compounds that have been receiving increasing attention, not only because of their structural interest but also owing to their varied applications (Davies *et al.*, 2008; Tian *et al.*, 2005). Several structures of triorganotin 2-hydroxybenzoates, such as (2-hydroxybenzoato)triphenyltin (Rauf *et al.*, 2008), (2-hydroxybenzoato)trimethyltin (Smith *et al.*, 1986), [5-(2-phenyl-1-diazenyl)-2-hydroxybenzoato]triphenyltin (Baul *et al.*, 2001) and (5-bromo-2-hydroxybenzoato)triphenyltin (Wang *et al.*, 2007), have been reported. As a continuation of these studies, the structure of the title compound is here described.

The coordination geometry of the Sn atom is that of a distorted tetrahedron (Fig. 1). The Sn···O1 separation of 2.962 (2) Å indicates there is a weak interaction between these atoms, which distorts the tetrahedral geometry by opening up the C1—Sn1—C7 angle to 116.2 (2)° and reducing the O2—Sn1—C13 angle to 94.9 (2)°. The monodentate mode of coordination of the benzoate group is reflected in the disparate O1—C19 and O2—C19 bond lengths of 1.227 (7) and 1.294 (7) Å, respectively. Bond dimensions around Sn atom are similar to those found in the structures of tricyclohexyltin benzoates such as tricyclohexyl(4-hydroxylbenzoato)tin (Song *et al.*, 2002) and tricyclohexyl[2-(2-(2-hydroxy-5-methylphenyl)-1-diazenyl)benzoato]tin (Willem *et al.*, 1998). An intramolecular O—H···O hydrogen bond is found between the carboxyl O1 atom and the phenolic hydroxy group (Fig. 1, Table 1).

### Experimental

Tricyclohexyltin hydroxide (1.05 g, 1 mmol) and 2-hydroxy-3,5-dibromobenzoic acid (0.77 g, 2 mmol) in toluene (50 ml) were refluxed for 5 h with azeotropic removal of water *via* a Dean–Stark trap. The resulting clear solution was evaporated under reduced pressure. The white solid obtained was purified by recrystallization from ethanol, and crystals of the title compound were obtained from a chloroform–hexane (1:1, *v*/*v*) solution by slow evaporation at room temperature (yield 74%, m.p. 392–393 K). Analysis, found: C 45.28, H 5.47%; calculated for C_25_H_36_Br_2_O_3_Sn: C 45.18, H 5.33%.

### Refinement

The bonds C15—C16 and C16—C17 were restrained to 1.52 Å. Atoms C3, C9 and C17 were also restrained to be approximately isotropic using the *ISOR* instruction in *SHELXL97* (Sheldrick, 2008). H atoms were placed in calculated positions (C—H = 0.93–0.98 Å and O—H = 0.82 Å) and refined as riding to their carrier atoms with *U*_iso_(H) = 1.2*U*_eq_(carrier C) and *U*_iso_(H3) = 1.5*U*_eq_(O3).

### Crystal data

**Table:**

[Sn(C_6_H_11_)_3_(C_7_H_3_Br_2_O_3_)]	*F*(000) = 1320
*M**_r_* = 663.05	*D*_x_ = 1.637 Mg m^−^^3^
Monoclinic, *P*2_1_/*c*	Melting point: 392 K
Hall symbol: -P 2ybc	Mo *K*α radiation, λ = 0.71073 Å
*a* = 9.4912 (13) Å	Cell parameters from 3561 reflections
*b* = 17.640 (2) Å	θ = 2.4–21.4°
*c* = 18.0655 (18) Å	µ = 3.94 mm^−^^1^
β = 117.200 (5)°	*T* = 295 K
*V* = 2690.1 (5) Å^3^	Block, colourless
*Z* = 4	0.20 × 0.20 × 0.10 mm

### Data collection

**Table:**

Bruker SMART APEX area-detector diffractometer	5000 independent reflections
Radiation source: fine-focus sealed tube	3416 reflections with *I* > 2σ(*I*)
graphite	*R*_int_ = 0.050
ω and φ scans	θ_max_ = 25.5°, θ_min_ = 1.7°
Absorption correction: multi-scan (*SADABS*; Bruker, 2002)	*h* = −11→11
*T*_min_ = 0.506, *T*_max_ = 0.694	*k* = −21→21
19711 measured reflections	*l* = −21→21

### Refinement

**Table:**

Refinement on *F*^2^	Primary atom site location: structure-invariant direct methods
Least-squares matrix: full	Secondary atom site location: difference Fourier map
*R*[*F*^2^ > 2σ(*F*^2^)] = 0.048	Hydrogen site location: inferred from neighbouring sites
*wR*(*F*^2^) = 0.136	H-atom parameters constrained
*S* = 1.03	*w* = 1/[σ^2^(*F*_o_^2^) + (0.0654*P*)^2^ + 2.5252*P*] where *P* = (*F*_o_^2^ + 2*F*_c_^2^)/3
5000 reflections	(Δ/σ)_max_ < 0.001
280 parameters	Δρ_max_ = 1.18 e Å^−^^3^
20 restraints	Δρ_min_ = −0.93 e Å^−^^3^
0 constraints	

### Fractional atomic coordinates and isotropic or equivalent isotropic displacement parameters (Å^2^)

**Table:**

	*x*	*y*	*z*	*U*_iso_*/*U*_eq_	
Sn1	−0.02284 (5)	−0.03487 (2)	0.17370 (2)	0.05127 (16)	
Br1	−0.05150 (12)	−0.16443 (5)	0.55475 (5)	0.0916 (3)	
Br2	−0.41868 (12)	0.10396 (7)	0.46748 (6)	0.1157 (4)	
O1	−0.2363 (6)	0.0418 (3)	0.2289 (3)	0.0745 (13)	
O2	−0.0590 (5)	−0.0504 (2)	0.2783 (2)	0.0581 (11)	
O3	−0.3647 (6)	0.0922 (3)	0.3169 (3)	0.0827 (15)	
H3	−0.3468	0.0897	0.2767	0.124*	
C1	−0.2443 (7)	−0.0635 (4)	0.0695 (3)	0.0531 (14)	
H1	−0.3199	−0.0234	0.0640	0.064*	
C2	−0.3114 (9)	−0.1376 (4)	0.0826 (5)	0.088 (2)	
H2A	−0.3320	−0.1330	0.1303	0.105*	
H2B	−0.2345	−0.1779	0.0940	0.105*	
C3	−0.4648 (11)	−0.1577 (5)	0.0055 (6)	0.111 (3)	
H3A	−0.4961	−0.2086	0.0118	0.134*	
H3B	−0.5476	−0.1234	0.0018	0.134*	
C4	−0.4523 (11)	−0.1535 (6)	−0.0729 (6)	0.112 (3)	
H4A	−0.5565	−0.1614	−0.1192	0.135*	
H4B	−0.3843	−0.1943	−0.0737	0.135*	
C5	−0.3877 (9)	−0.0797 (6)	−0.0853 (5)	0.095 (3)	
H5A	−0.3737	−0.0818	−0.1352	0.113*	
H5B	−0.4608	−0.0388	−0.0915	0.113*	
C6	−0.2316 (9)	−0.0658 (5)	−0.0107 (4)	0.086 (2)	
H6A	−0.1887	−0.0180	−0.0177	0.103*	
H6B	−0.1582	−0.1055	−0.0071	0.103*	
C7	0.0607 (8)	0.0785 (3)	0.1761 (4)	0.0616 (17)	
H7	0.1360	0.0750	0.1531	0.074*	
C8	−0.0656 (9)	0.1329 (4)	0.1204 (5)	0.081 (2)	
H8A	−0.1512	0.1334	0.1357	0.097*	
H8B	−0.1083	0.1157	0.0632	0.097*	
C9	−0.0006 (12)	0.2130 (5)	0.1271 (6)	0.105 (3)	
H9A	0.0668	0.2146	0.0998	0.126*	
H9B	−0.0883	0.2475	0.0978	0.126*	
C10	0.0909 (11)	0.2395 (4)	0.2135 (6)	0.100 (3)	
H10A	0.0200	0.2456	0.2383	0.120*	
H10B	0.1362	0.2888	0.2133	0.120*	
C11	0.2194 (11)	0.1868 (4)	0.2650 (6)	0.099 (3)	
H11A	0.2720	0.2051	0.3219	0.118*	
H11B	0.2971	0.1848	0.2441	0.118*	
C12	0.1548 (9)	0.1075 (4)	0.2636 (4)	0.079 (2)	
H12A	0.2422	0.0732	0.2942	0.095*	
H12B	0.0877	0.1084	0.2911	0.095*	
C13	0.1573 (8)	−0.1191 (4)	0.2083 (4)	0.0667 (18)	
H13	0.1141	−0.1665	0.2178	0.080*	
C14	0.3015 (12)	−0.0985 (6)	0.2890 (6)	0.128 (4)	
H14A	0.3449	−0.0511	0.2811	0.154*	
H14B	0.2685	−0.0904	0.3319	0.154*	
C15	0.4288 (14)	−0.1575 (7)	0.3182 (7)	0.156 (5)	
H15A	0.5200	−0.1398	0.3678	0.188*	
H15B	0.3905	−0.2037	0.3320	0.188*	
C16	0.4755 (12)	−0.1732 (8)	0.2513 (8)	0.162 (5)	
H16A	0.5512	−0.2146	0.2689	0.194*	
H16B	0.5280	−0.1287	0.2439	0.194*	
C17	0.3380 (14)	−0.1934 (7)	0.1690 (8)	0.157 (5)	
H17A	0.2955	−0.2421	0.1739	0.189*	
H17B	0.3752	−0.1983	0.1273	0.189*	
C18	0.2065 (12)	−0.1340 (6)	0.1403 (6)	0.125 (4)	
H18A	0.2435	−0.0873	0.1268	0.150*	
H18B	0.1158	−0.1517	0.0904	0.150*	
C19	−0.1639 (8)	−0.0060 (4)	0.2822 (4)	0.0560 (15)	
C20	−0.1898 (7)	−0.0157 (3)	0.3567 (3)	0.0498 (14)	
C21	−0.1200 (7)	−0.0745 (4)	0.4126 (4)	0.0539 (15)	
H21	−0.0564	−0.1092	0.4031	0.065*	
C22	−0.1430 (8)	−0.0821 (4)	0.4808 (4)	0.0580 (16)	
C23	−0.2333 (8)	−0.0288 (4)	0.4974 (4)	0.0690 (19)	
H23	−0.2459	−0.0329	0.5454	0.083*	
C24	−0.3028 (8)	0.0290 (4)	0.4433 (4)	0.0684 (19)	
C25	−0.2866 (8)	0.0358 (4)	0.3710 (4)	0.0625 (17)	

### Atomic displacement parameters (Å^2^)

**Table:**

	*U*^11^	*U*^22^	*U*^33^	*U*^12^	*U*^13^	*U*^23^
Sn1	0.0568 (3)	0.0573 (3)	0.0429 (2)	−0.0012 (2)	0.0256 (2)	0.00025 (19)
Br1	0.1340 (8)	0.0825 (5)	0.0670 (5)	−0.0002 (5)	0.0536 (5)	0.0160 (4)
Br2	0.1133 (8)	0.1531 (9)	0.0973 (7)	0.0402 (7)	0.0625 (6)	−0.0059 (6)
O1	0.079 (3)	0.093 (4)	0.052 (3)	0.026 (3)	0.031 (2)	0.024 (2)
O2	0.068 (3)	0.068 (3)	0.045 (2)	0.006 (2)	0.031 (2)	0.0053 (19)
O3	0.081 (3)	0.101 (4)	0.064 (3)	0.037 (3)	0.031 (3)	0.013 (3)
C1	0.051 (4)	0.062 (4)	0.042 (3)	0.004 (3)	0.018 (3)	0.001 (3)
C2	0.073 (5)	0.082 (5)	0.081 (5)	−0.016 (4)	0.012 (4)	0.015 (4)
C3	0.094 (6)	0.108 (6)	0.107 (6)	−0.035 (5)	0.024 (5)	0.012 (5)
C4	0.085 (6)	0.129 (8)	0.081 (6)	0.011 (6)	0.002 (5)	−0.046 (6)
C5	0.066 (5)	0.155 (9)	0.051 (4)	−0.007 (5)	0.017 (4)	−0.025 (5)
C6	0.072 (5)	0.135 (7)	0.050 (4)	0.000 (5)	0.029 (4)	−0.008 (4)
C7	0.075 (5)	0.060 (4)	0.064 (4)	−0.011 (3)	0.044 (4)	−0.005 (3)
C8	0.085 (5)	0.075 (5)	0.070 (5)	0.008 (4)	0.024 (4)	0.018 (4)
C9	0.118 (6)	0.085 (5)	0.100 (6)	−0.004 (5)	0.040 (5)	0.031 (4)
C10	0.129 (8)	0.063 (5)	0.108 (7)	0.008 (5)	0.055 (6)	0.004 (5)
C11	0.110 (7)	0.068 (5)	0.097 (6)	−0.016 (5)	0.029 (5)	−0.012 (5)
C12	0.090 (5)	0.058 (4)	0.067 (5)	−0.005 (4)	0.017 (4)	−0.006 (3)
C13	0.064 (4)	0.065 (4)	0.080 (5)	0.010 (3)	0.040 (4)	0.006 (4)
C14	0.113 (7)	0.138 (8)	0.080 (6)	0.068 (7)	−0.003 (5)	−0.012 (6)
C15	0.123 (9)	0.152 (11)	0.138 (10)	0.069 (8)	0.011 (8)	0.000 (8)
C16	0.079 (7)	0.166 (12)	0.223 (15)	0.022 (7)	0.054 (9)	−0.046 (10)
C17	0.151 (7)	0.163 (7)	0.171 (7)	0.039 (6)	0.085 (6)	−0.036 (6)
C18	0.118 (8)	0.169 (10)	0.093 (7)	0.061 (7)	0.051 (6)	−0.007 (6)
C19	0.056 (4)	0.068 (4)	0.044 (4)	−0.002 (3)	0.023 (3)	−0.002 (3)
C20	0.048 (3)	0.061 (4)	0.040 (3)	−0.005 (3)	0.020 (3)	−0.007 (3)
C21	0.050 (4)	0.062 (4)	0.049 (3)	−0.012 (3)	0.023 (3)	−0.004 (3)
C22	0.063 (4)	0.069 (4)	0.042 (3)	−0.012 (3)	0.025 (3)	−0.001 (3)
C23	0.065 (4)	0.099 (6)	0.047 (4)	−0.014 (4)	0.029 (3)	−0.002 (4)
C24	0.062 (4)	0.096 (5)	0.054 (4)	0.007 (4)	0.032 (3)	−0.006 (4)
C25	0.054 (4)	0.081 (5)	0.050 (4)	0.003 (4)	0.022 (3)	−0.003 (3)

### Geometric parameters (Å, °)

**Table:**

Sn1—O2	2.086 (4)	C9—H9B	0.9700
Sn1—C13	2.132 (6)	C10—C11	1.477 (11)
Sn1—C7	2.144 (6)	C10—H10A	0.9700
Sn1—C1	2.144 (6)	C10—H10B	0.9700
Br1—C22	1.894 (6)	C11—C12	1.523 (10)
Br2—C24	1.894 (7)	C11—H11A	0.9700
O1—C19	1.227 (7)	C11—H11B	0.9700
O2—C19	1.294 (7)	C12—H12A	0.9700
O3—C25	1.353 (8)	C12—H12B	0.9700
O3—H3	0.8200	C13—C14	1.519 (11)
C1—C6	1.508 (9)	C13—C18	1.524 (10)
C1—C2	1.520 (9)	C13—H13	0.9800
C1—H1	0.9800	C14—C15	1.496 (12)
C2—C3	1.527 (11)	C14—H14A	0.9700
C2—H2A	0.9700	C14—H14B	0.9700
C2—H2B	0.9700	C15—C16	1.492 (9)
C3—C4	1.476 (13)	C15—H15A	0.9700
C3—H3A	0.9700	C15—H15B	0.9700
C3—H3B	0.9700	C16—C17	1.506 (9)
C4—C5	1.499 (13)	C16—H16A	0.9700
C4—H4A	0.9700	C16—H16B	0.9700
C4—H4B	0.9700	C17—C18	1.527 (13)
C5—C6	1.498 (10)	C17—H17A	0.9700
C5—H5A	0.9700	C17—H17B	0.9700
C5—H5B	0.9700	C18—H18A	0.9700
C6—H6A	0.9700	C18—H18B	0.9700
C6—H6B	0.9700	C19—C20	1.484 (8)
C7—C12	1.505 (9)	C20—C21	1.387 (8)
C7—C8	1.506 (9)	C20—C25	1.398 (8)
C7—H7	0.9800	C21—C22	1.353 (8)
C8—C9	1.525 (10)	C21—H21	0.9300
C8—H8A	0.9700	C22—C23	1.394 (9)
C8—H8B	0.9700	C23—C24	1.357 (9)
C9—C10	1.473 (12)	C23—H23	0.9300
C9—H9A	0.9700	C24—C25	1.389 (9)
			
O2—Sn1—C13	94.9 (2)	C10—C11—H11A	109.4
O2—Sn1—C7	108.4 (2)	C12—C11—H11A	109.4
C13—Sn1—C7	113.9 (3)	C10—C11—H11B	109.4
O2—Sn1—C1	105.2 (2)	C12—C11—H11B	109.4
C13—Sn1—C1	115.2 (3)	H11A—C11—H11B	108.0
C7—Sn1—C1	116.2 (2)	C7—C12—C11	111.8 (6)
C19—O2—Sn1	114.8 (4)	C7—C12—H12A	109.3
C25—O3—H3	109.5	C11—C12—H12A	109.3
C6—C1—C2	110.5 (6)	C7—C12—H12B	109.3
C6—C1—Sn1	111.9 (4)	C11—C12—H12B	109.3
C2—C1—Sn1	112.2 (4)	H12A—C12—H12B	107.9
C6—C1—H1	107.3	C14—C13—C18	109.9 (7)
C2—C1—H1	107.3	C14—C13—Sn1	111.4 (5)
Sn1—C1—H1	107.3	C18—C13—Sn1	112.6 (5)
C1—C2—C3	110.4 (6)	C14—C13—H13	107.6
C1—C2—H2A	109.6	C18—C13—H13	107.6
C3—C2—H2A	109.6	Sn1—C13—H13	107.6
C1—C2—H2B	109.6	C15—C14—C13	113.6 (8)
C3—C2—H2B	109.6	C15—C14—H14A	108.8
H2A—C2—H2B	108.1	C13—C14—H14A	108.8
C4—C3—C2	113.8 (8)	C15—C14—H14B	108.8
C4—C3—H3A	108.8	C13—C14—H14B	108.8
C2—C3—H3A	108.8	H14A—C14—H14B	107.7
C4—C3—H3B	108.8	C16—C15—C14	109.5 (9)
C2—C3—H3B	108.8	C16—C15—H15A	109.8
H3A—C3—H3B	107.7	C14—C15—H15A	109.8
C3—C4—C5	113.8 (7)	C16—C15—H15B	109.8
C3—C4—H4A	108.8	C14—C15—H15B	109.8
C5—C4—H4A	108.8	H15A—C15—H15B	108.2
C3—C4—H4B	108.8	C15—C16—C17	113.5 (9)
C5—C4—H4B	108.8	C15—C16—H16A	108.9
H4A—C4—H4B	107.7	C17—C16—H16A	108.9
C6—C5—C4	108.2 (8)	C15—C16—H16B	108.9
C6—C5—H5A	110.1	C17—C16—H16B	108.9
C4—C5—H5A	110.1	H16A—C16—H16B	107.7
C6—C5—H5B	110.1	C16—C17—C18	112.2 (9)
C4—C5—H5B	110.1	C16—C17—H17A	109.2
H5A—C5—H5B	108.4	C18—C17—H17A	109.2
C5—C6—C1	112.8 (6)	C16—C17—H17B	109.2
C5—C6—H6A	109.0	C18—C17—H17B	109.2
C1—C6—H6A	109.0	H17A—C17—H17B	107.9
C5—C6—H6B	109.0	C13—C18—C17	110.4 (8)
C1—C6—H6B	109.0	C13—C18—H18A	109.6
H6A—C6—H6B	107.8	C17—C18—H18A	109.6
C12—C7—C8	113.2 (6)	C13—C18—H18B	109.6
C12—C7—Sn1	112.0 (4)	C17—C18—H18B	109.6
C8—C7—Sn1	114.0 (5)	H18A—C18—H18B	108.1
C12—C7—H7	105.6	O1—C19—O2	122.8 (6)
C8—C7—H7	105.6	O1—C19—C20	121.7 (6)
Sn1—C7—H7	105.6	O2—C19—C20	115.5 (6)
C7—C8—C9	111.6 (7)	C21—C20—C25	119.1 (6)
C7—C8—H8A	109.3	C21—C20—C19	121.7 (6)
C9—C8—H8A	109.3	C25—C20—C19	119.2 (6)
C7—C8—H8B	109.3	C22—C21—C20	121.1 (6)
C9—C8—H8B	109.3	C22—C21—H21	119.5
H8A—C8—H8B	108.0	C20—C21—H21	119.5
C10—C9—C8	113.6 (7)	C21—C22—C23	120.0 (6)
C10—C9—H9A	108.9	C21—C22—Br1	120.4 (5)
C8—C9—H9A	108.9	C23—C22—Br1	119.6 (5)
C10—C9—H9B	108.9	C24—C23—C22	119.8 (6)
C8—C9—H9B	108.9	C24—C23—H23	120.1
H9A—C9—H9B	107.7	C22—C23—H23	120.1
C9—C10—C11	112.6 (7)	C23—C24—C25	121.1 (6)
C9—C10—H10A	109.1	C23—C24—Br2	120.1 (5)
C11—C10—H10A	109.1	C25—C24—Br2	118.8 (5)
C9—C10—H10B	109.1	O3—C25—C24	118.9 (6)
C11—C10—H10B	109.1	O3—C25—C20	122.3 (6)
H10A—C10—H10B	107.8	C24—C25—C20	118.8 (6)
C10—C11—C12	111.0 (7)		
			
C13—Sn1—O2—C19	178.6 (5)	O2—Sn1—C13—C18	172.8 (6)
C7—Sn1—O2—C19	61.4 (5)	C7—Sn1—C13—C18	−74.6 (7)
C1—Sn1—O2—C19	−63.6 (5)	C1—Sn1—C13—C18	63.5 (7)
O2—Sn1—C1—C6	−172.0 (5)	C18—C13—C14—C15	−57.2 (12)
C13—Sn1—C1—C6	−69.0 (6)	Sn1—C13—C14—C15	177.3 (8)
C7—Sn1—C1—C6	68.0 (6)	C13—C14—C15—C16	55.9 (14)
O2—Sn1—C1—C2	−47.2 (5)	C14—C15—C16—C17	−53.6 (15)
C13—Sn1—C1—C2	55.9 (6)	C15—C16—C17—C18	53.7 (16)
C7—Sn1—C1—C2	−167.2 (5)	C14—C13—C18—C17	53.9 (11)
C6—C1—C2—C3	−51.7 (9)	Sn1—C13—C18—C17	178.8 (7)
Sn1—C1—C2—C3	−177.3 (6)	C16—C17—C18—C13	−53.0 (14)
C1—C2—C3—C4	49.6 (11)	Sn1—O2—C19—O1	−0.3 (8)
C2—C3—C4—C5	−52.3 (11)	Sn1—O2—C19—C20	−179.4 (4)
C3—C4—C5—C6	54.8 (10)	O1—C19—C20—C21	173.5 (6)
C4—C5—C6—C1	−58.5 (10)	O2—C19—C20—C21	−7.4 (8)
C2—C1—C6—C5	58.7 (9)	O1—C19—C20—C25	−6.7 (9)
Sn1—C1—C6—C5	−175.5 (6)	O2—C19—C20—C25	172.4 (6)
O2—Sn1—C7—C12	30.2 (6)	C25—C20—C21—C22	−0.6 (9)
C13—Sn1—C7—C12	−74.0 (6)	C19—C20—C21—C22	179.1 (6)
C1—Sn1—C7—C12	148.5 (5)	C20—C21—C22—C23	−2.4 (9)
O2—Sn1—C7—C8	−99.9 (5)	C20—C21—C22—Br1	178.2 (4)
C13—Sn1—C7—C8	155.9 (5)	C21—C22—C23—C24	2.5 (10)
C1—Sn1—C7—C8	18.3 (6)	Br1—C22—C23—C24	−178.1 (5)
C12—C7—C8—C9	48.3 (9)	C22—C23—C24—C25	0.5 (10)
Sn1—C7—C8—C9	177.9 (6)	C22—C23—C24—Br2	−177.4 (5)
C7—C8—C9—C10	−49.3 (11)	C23—C24—C25—O3	176.7 (6)
C8—C9—C10—C11	53.8 (11)	Br2—C24—C25—O3	−5.3 (9)
C9—C10—C11—C12	−55.8 (11)	C23—C24—C25—C20	−3.5 (10)
C8—C7—C12—C11	−51.7 (9)	Br2—C24—C25—C20	174.5 (5)
Sn1—C7—C12—C11	177.7 (6)	C21—C20—C25—O3	−176.7 (6)
C10—C11—C12—C7	54.6 (10)	C19—C20—C25—O3	3.6 (9)
O2—Sn1—C13—C14	−63.2 (7)	C21—C20—C25—C24	3.5 (9)
C7—Sn1—C13—C14	49.5 (7)	C19—C20—C25—C24	−176.3 (6)
C1—Sn1—C13—C14	−172.5 (6)		

### Hydrogen-bond geometry (Å, °)

**Table:**

*D*—H···*A*	*D*—H	H···*A*	*D*···*A*	*D*—H···*A*
O3—H3···O1	0.82	1.84	2.564 (7)	147.
